# Effect of plasma sericin glutaraldehyde treatments on the low stress mechanical properties of micro denier polyester/cotton blended fabric

**DOI:** 10.1038/s41598-022-13661-9

**Published:** 2022-06-08

**Authors:** M. Rajalakshmi, S. Kubera Sampath Kumar, D. Vasanth Kumar, M. Siva Jagadish Kumar, C. Prakash

**Affiliations:** 1 Department of Costume Design and Fashion, VET Institute of Arts and Science College, Thindal, Erode, Tamil Nadu 638012 India; 2 Department of Chemical Engineering (Textile Technology), Vignan’s Foundation for Science, Technology and Research (Deemed to be University), Vadlamudi, Guntur, Andhra Pradesh 522213 India; 3 VIT Fashion Institute of Technology (VFIT), Vellore Institute of Technology (VIT), Chennai, Chennai, Tamil Nadu 600 127 India; 4 Department of Handloom and Textile Technology, Indian Institute of Handloom Technology, Ministry of Textiles, Govt. of India, Fulia Colony, Shantipur, Nadia, West Bengal 741 402 India

**Keywords:** Engineering, Materials science

## Abstract

This study aimed to see how different treatments affect the low-stress mechanical properties of micro-denier polyester/cotton (MDP/C—65/35) fabrics. This blend was chosen for the study because it is the most popular blend used in polyester/cotton blended material. The results of fabric properties treated with sericin revealed that fabrics treated with sericin and glutaraldehyde as a cross-linking agent had higher bending rigidity, regardless of how it was tested. Concerning the blend fabrics, it was noticed that there was deterioration in tensile resilience following sericin treatment. Shear rigidity, accompanied by shear hysteresis, showed an increase in sericin-treated fabrics. Compression properties were affected by the treatment, and in general, the fabric suffered deterioration in those the samples were hard. Surface properties such as coefficient of friction, mean deviation of friction and mean deviation of surface contour were found to be higher than those of the control and sericin-treated fabrics in a few cases.

## Introduction

Innovative production techniques are the need of the hour for the textile industries to improve product quality and functional properties. In recent years, microfibers are emerging in the textile industry for various applications and similarly, manmade fibers are being upgraded to make them superior to natural fibers in appearance, physical and comfort properties^1,2^.The blending of microdenier polyester fibers (MDP/C) along with cotton fibers has become popular because it gives a valuable combination of properties to both fibers^3^. It is evidenced that micro denier polyester fibers blended with cotton fibers results in enhanced fabric properties and contributes to high strength, good wicking, moisture absorbency, air permeability properties and environmental friendliness^4,5^. In the creation of innovative functional textiles, manipulating the surface properties of textile materials is critical^6^. The functional characteristics of fibers are modified via plasma surface treatment. Plasma uses less water and energy while causing very little fiber damage, making it a very desirable technique. It will be utilised in fabric preparation, dyeing, and finishing procedures to improve the quality of textile products^7–9^.

Bio modification of synthetic fibers is a relatively new and promising method. In general, synthetic materials have been thought to be resistant to biological deterioration. Bio modification, on the other hand, has recently been proved to be capable of hydrolyzing synthetic materials, resulting in antimicrobial textiles by various research groups. Because of their potential to provide individuals with high-quality life and safety benefits, these have piqued the interest of both academic research and industry.

Sericin can be cross-linked, copolymerized, and combined with other polymers to create new biodegradable materials with better characteristics^10,11^. In addition, sericin can also be used as an improving reagent or a coating material for natural and synthetic fibers and fabrics. By employing dimethylol dihydroxy ethylene urea (DMDHEU) as a cross-linking agent, sericin has been discovered to improve moisture regain and water retention while also lowering electrical resistance in cotton fibers^12^. Gulrajani et. al.^13^ conducted a study on the application of sericin on polyester fabric (pre-treated with sodium hydroxide) along with glutaraldehyde. They concluded that there was a noticeable improvement in the moisture absorption, antimicrobial activity, antistatic and Ultra Violet (UV) resistant properties of the sericin-treated fabric^14,15^. Recently, the silkworm has been used as a bio factory for the production of useful protein through its silk gland, and silk has become a valuable biomaterial for diverse pragmatic applications^16^. In textiles, it has been used for the functionalization of natural and synthetic fibers to improve water absorbency and smooth surfaces. Polyester fibers have been modified with sericin by cross-linking with glycerol polyglycidyl ether and diethylene triamine^17^.

Although little research work has been carried out on the application of both sericin and plasma on textiles, there is a need for intensive studies, especially those that explore the variables that a combination of this type offers. For example, aspects such as pre-treatment of the textile substrates prior to the application of sericin and plasma, sericin concentration, post-treatment of sericin on plasma-treated textiles, the process conditions that ensure better fixation, method of application of sericin to the textile substrate and so on require careful investigation. A review of past work on the application of sericin to fabrics such as polyester and cotton has revealed that information on the low-stress mechanical properties of such fabric is sparse. In the evolution of this research, it became apparent that the application of sericin on polyester blended fabrics needed examination through the development of improved instrumentation for determining low-stress mechanical properties.

## Materials and methods

### Selection of fiber samples

MDP/C blended yarn (19.5 Tex) produced in the ratios of 65/35. Using a sample loom, weave fabric samples were produced from the yarn samples stated above. The loom settings were controlled to obtain 147 cm width of fabric with construction particulars of 26.77 Ends Per cm and 26.77 Picks Per cm. A portion of the fabric samples was preserved to serve as the ‘control sample’.

### Sericin from yellow cocoons: extraction and purification

As per the standard material to liquor (M:L) ratioof 1:40, silk cocoons were cleaned, chopped and treated in a steam bath with sodium carbonate (1.06%) and sodium bicarbonate (0.84%) at 100 °C for 60 min. Filtration was used to remove the insoluble fibroin. Three litres of ice-cold ethanol were used to precipitate the resultant liquid, which was then incubated for an hour at 4 °C. After incubation, the fluid was centrifuged for 15 min at 5000 rpm. The pellets that resulted were then collected and dissolved in distilled water. The developed fabric samples were finished with the prepared sericin solution^18^.

### Glutaraldehyde finishing of fabric samples

A 2 dip/2 nip technique was used to treat MDP/C blended fabrics with glutaraldehyde (10 g/L), magnesium chloride (10 g/L), and acetic acid (0.1%) in a padding mangle. The padded materials were dried and cured for 3 min at 150°.

### Finishing of fabric samples with sericin

Micro denier polyester/cotton blended fabrics were scoured using Lissapol N to remove any finish and were treated with oxygen plasma. A 2dip/2nip procedure was used to pad the control and plasma-treated micro denier polyester/cotton blended fabrics with sericin solution (10 g/L) in a padding mangle (with 80°expression). For 3 min, the padded fabrics were dried and cured at 150 °C.

### Finishing of samples with sericin and glutaraldehyde

The solution was prepared by mixing sericin (10 g/L) with glutaraldehyde (10 ml/L), magnesium chloride (10 g/L), and acetic acid (0.1%). Then, this solution was applied to untreated micro denier polyester/cotton blended fabrics by the padding method. The padded fabric was dried for 3 min at 80 °C and then cured for 3 min at 150 °C.

### Oxygen plasma treatment of selected fabric samples

The fabric samples were treated with oxygen plasma using oxygen as the working gas. An HPVT Plasma System Model-PS was used. The airflow rate was 10 cm^3^/min, and the distance between the electrodes was 2 mm. The plasma pressure was maintained at 200 millibars, and the time of treatment was 20 s.

### Measurement of low-stress mechanical properties

The KES—FBinstrument measures various low-stress mechanical properties of fabrics i.e., low mechanical stress tensile, shear (KES-FB-1), bending (KES-FB-2), compression (KES-FB-3) and surface friction (KES-FB-4), mechanical properties that correspond to the fundamental deformation of fabrics in-hand manipulation. All the test methods performed as per KES – FB standard methods^19,20^,the fabric samples are designated as indicated in Table 1.

**Table 1:** Fabric finish details and designation.

Fabric number	Fabric description
1	Control
2	Glutaraldehyde treated
3	Sericin glutaraldehyde treated
4	Plasma treated
5	Plasma sericin glutaraldehyde treated

## Results and discussions

### Tensile properties

**Figure 1:**
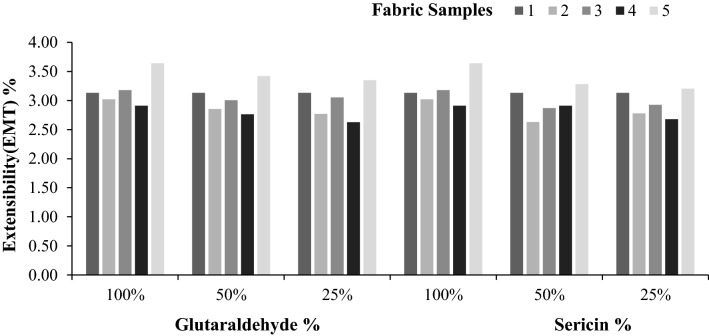
Extensibility (EMT%) of samples.

**Figure 2:**
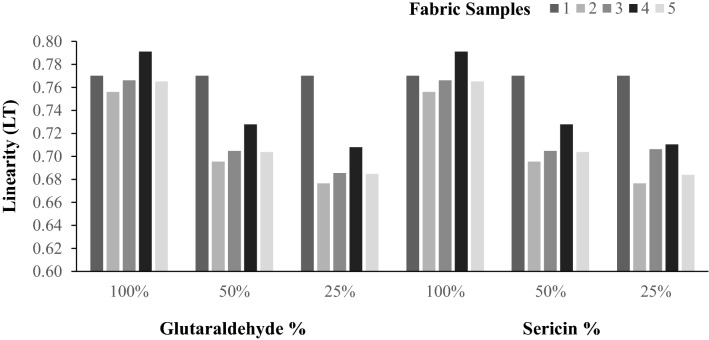
Linearity (LT) of samples.

**Figure 3:**
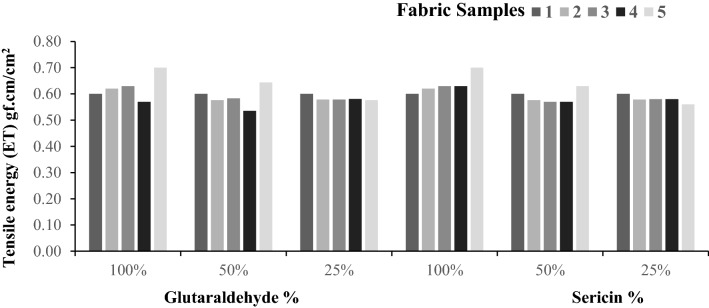
Tensile energy (ET) of sample.

**Figure 4:**
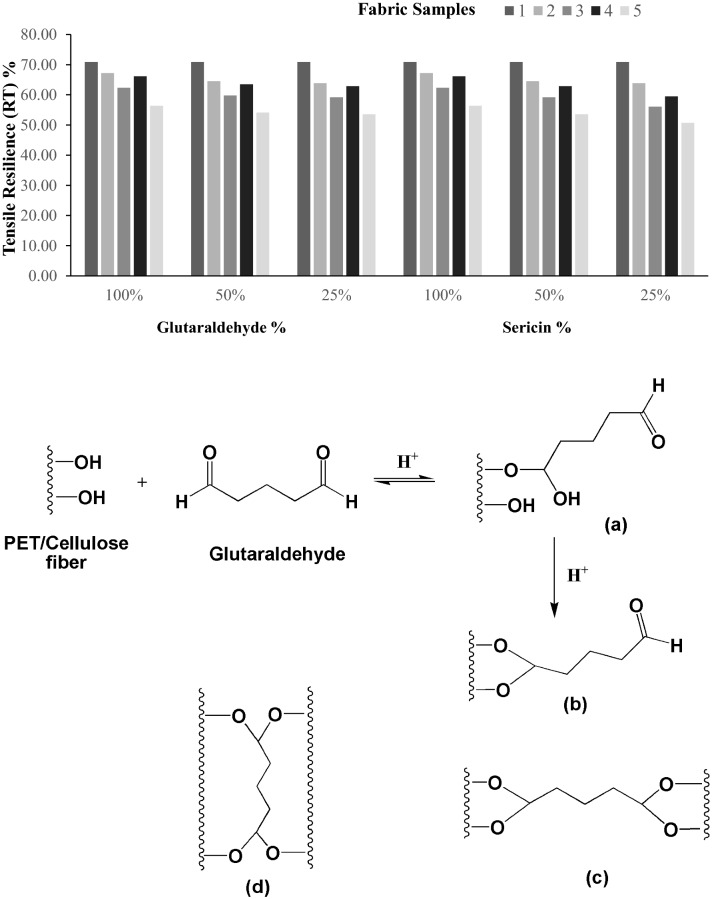
Tensile resilience (RT) % of samples. (**a**–**d**) Possible ways of fixation of glutaraldehyde on the fabric sample.

The linearity of the load extension curve (LT), tensile energy (ET), tensile resilience (RT), and elongation (EMT) is the low-stress tensile parameters that have been assessed, as represented graphically in Figs. 1, 2, 3, 4.

Figures 1 and 2 show the extensibility and linearity of the samples. Regarding extensibility (EMT), the statistical analysis shows that there is no significant difference. Because of this, the treatments have practically no effect on extensibility. The same comments hold good linearity of the load extension curve.

Figures 3 and 4 represent the tensile energy (ET) and tensile resilience (RT) values. In the case of tensile energy values, again, there is no significant difference. However, there is a significant difference between the values concerning tensile resilience. The fabric that has been applied with plasma following treatment with sericin and glutaraldehyde exhibits a significantly lower value in comparison to the control. The fixation of the sericin/glutaraldehyde on the fabric sample represented in Fig. 4a, and the sericin-treated fabric also shows a higher tensile resilience, which is due to sericin containing random coil α-structure with less β-sheet structure. It is soluble in hot water, and when the temperature drops, the random coil β-structure of the silk is transformed to α-sheet structure, which improves the viscoelastic nature of the silk, resulting in gel formation and giving the coated material increased elasticity and durability^21,22^.

### Bending properties

A higher fabric bending rigidity means stiff fabric and its drape are low. Similarly, too low a value of bending rigidity means that the fabric would be too flexible or supplied. For good drapability, a certain minimum bending rigidity is required. To impart higher bending rigidity (B) and hysteresis (2HB), it is necessary to subject the fabric to finishing treatment. These treatments increase the fabric rigidity and elastic recovery from bending. A larger value of 2HB means greater fabric inelasticity.

**Figure 5:**
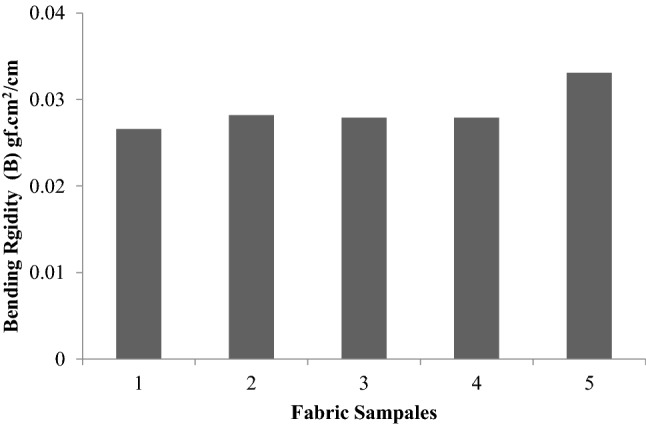
Bending rigidity (B) of samples.

**Figure 6:**
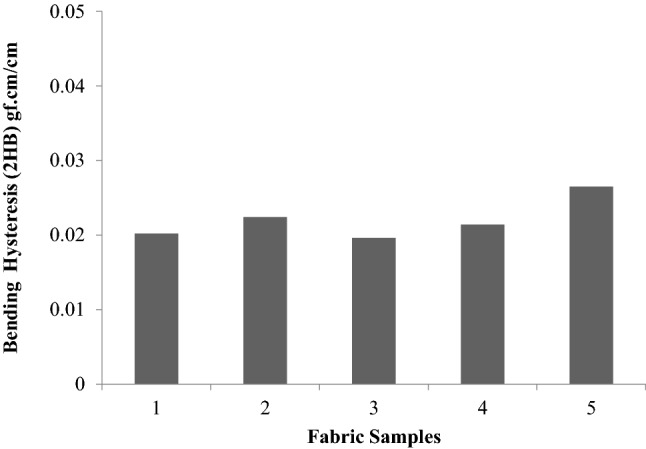
Bending hysteresis (2HB) of samples.

**Figure 7:**
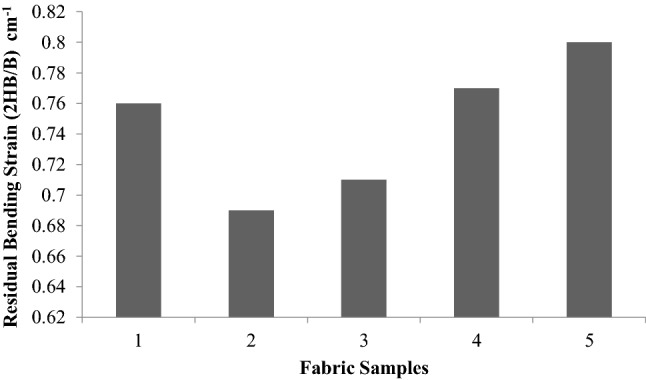
Residual bending strain (2HB/B).

Figures 5, 6, 7 show the values of bending rigidity, bending hysteresis and residual bending strain. Bending rigidity values do not show any significant difference between the treatments, as evidenced by the “t” test. Bending hysteresis also follow the same trend. There is no significant difference in the residual bending strain. However the treatment of plasma improves the adhesion, so MDC/P absorb more sericin glutaraldehyde on the surface of the sample (sample 5) when compared to the untreated samples, so the bending rigidity, residual bending strain value of the treated sample increased represented in Figs. 5, 6, 7. ^23,24^.

### Shear properties

Shear properties are considered to be important for successful draping and fabric forming qualities that are necessary for successful tailoring and garment wear. The shear properties were measured using a KES-FB instrument. The values measured are G (shear rigidity), shear hysteresis at a shear angle of 0.5 degrees (2HG) andshear angle of 5 degrees (2HG5).

**Figure 8:**
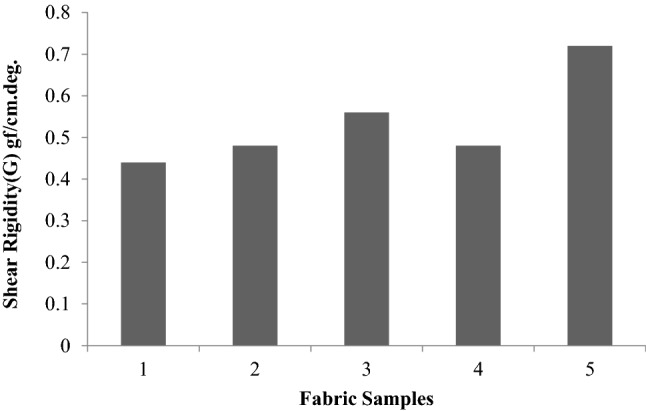
Shear rigidity (G) of fabrics.

Figure 8 shows the shear stiffness (G) of the fabrics. As far as the shear rigidity values are concerned, there is no evidence to show that the treatments have any effect on it. Except for the fabric treated with sericin following pre-treatment with plasma and glutaraldehyde (Sample 5), there was not much difference in other cases. This may be due to the stiffening action of the finishing agent since the combination of glutaraldehyde and sericin was used, which might have led to the stiffening of the fabric.

**Figure 9:**
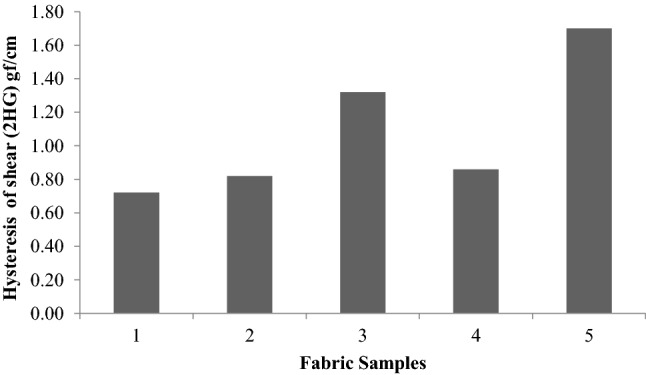
Hysteresis of shear (2HG) of fabrics.

Figure 9 shows the shear hysteresis at a shear angle of 0.5 degrees (2HG). The shear hysteresis also follows the same trend as the shear rigidity.

**Figure 10:**
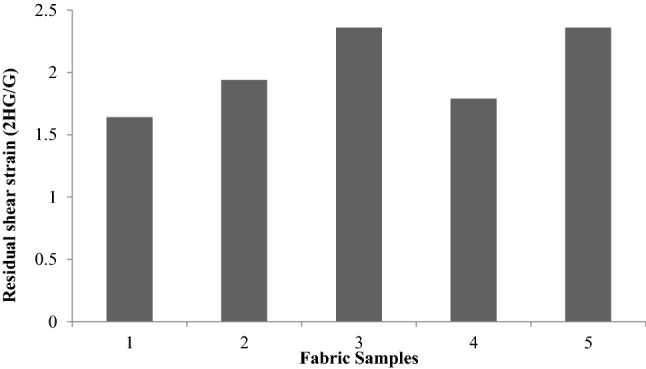
Residual shear strain (2HG/G).

Figure 10 shows the residual shear strain 2HG/G. With the exception of the sericin-glutaraldehyde-treated sample and plasma-sericin-glutaraldehyde-treated sample, the residual shear strain values are not significant. The residual shear strain 2HG/G is found to be highest in plasma- and sericin- and glutaraldehydetreated samples, which indicates that the recovery is less. This is due to the presence of sericin gum, which impedes the recovery of the fabrics from shear.

During the degumming process the random coil α-structure converted into β-pleated sheets act as the stiffening mechanism in the silk^25^.

**Figure 11:**
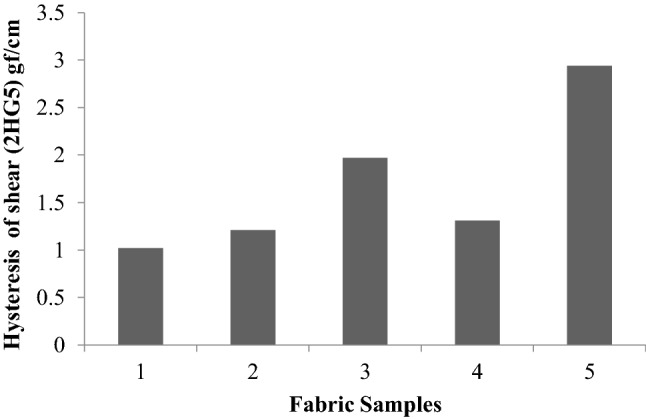
Hysteresis of shear (2HG5) of fabrics.

Figure 11 shows the hysteresis of shear at a shear angle of 5 degrees (2HG5). This follows the same trend as shear hysteresis at 0.5°. In particular, sericin and glutaraldehyde treatment show a pronounced effect with respect to shear hysteresis.

**Figure 12:**
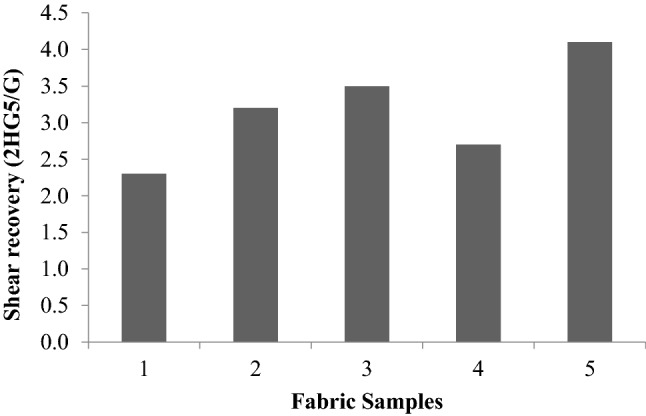
Shear recovery (2HG5/G).

Figure 12 represents the shear recovery of the fabrics. The values are found to be significant in most of the cases. However, the plasma-treated sample was similar to the control fabric.

### Compression properties

**Figure 13:**
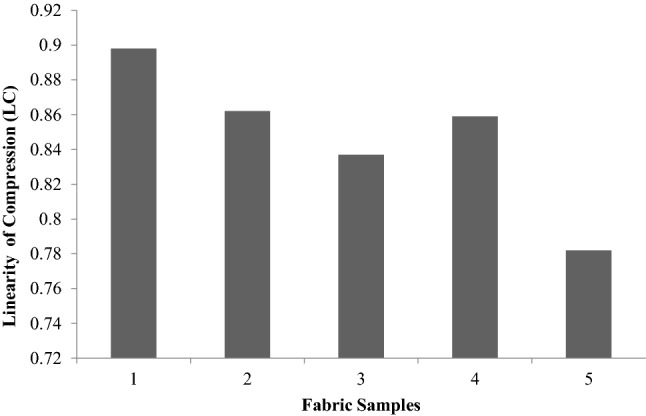
Linearity of compression (LC) of fabrics.

**Figure 14:**
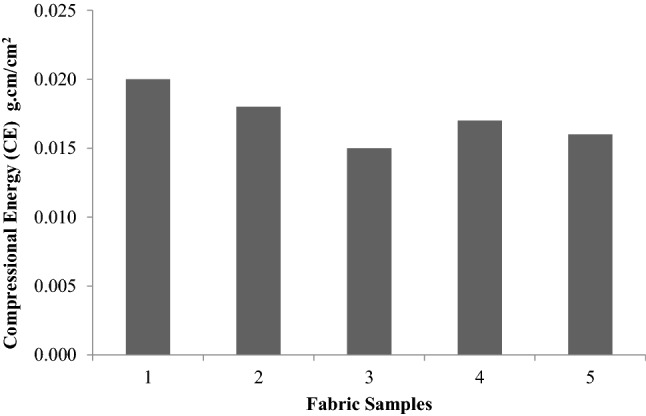
Compressional energy (CE) of fabrics.

**Figure 15:**
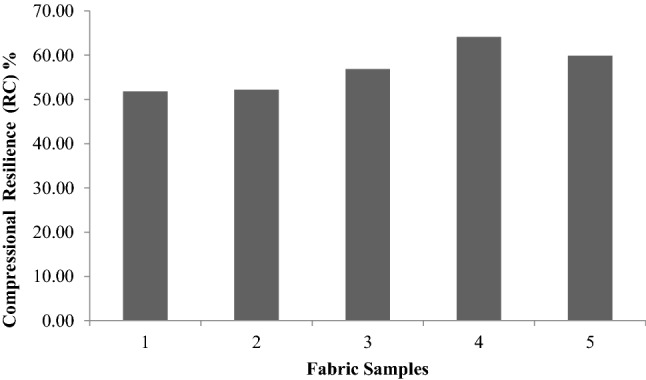
Compressional resilience (RC) % of fabrics.

**Figure 16:**
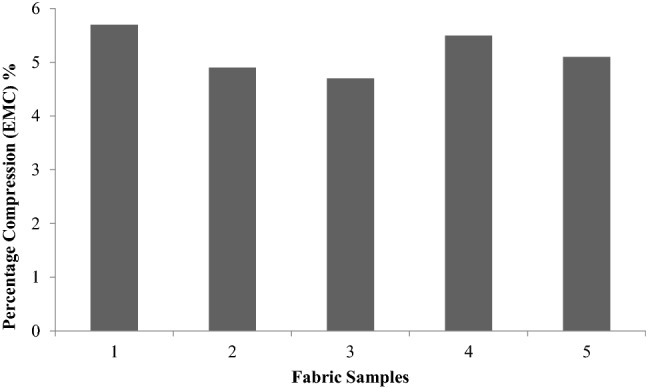
Percentage compression.

Unlike the tensile, bending, shear and surface properties, compression properties merit special consideration, as they are single values such as drapes. This type of compression refers to transverse compression.Compression properties provide an idea about the handling of the fabric. LC, CE and RC are defined in the same way as LT, ET and RT, respectively. Figures 13, 14, 15, 16 are thevalues obtained from the compression test of the fabrics. LC values are a measure of the linearity of the compression curve. CE describes fabric compressive toughness. RC indicates the rate of fabric elastic recovery. Among the three parameters that are used to represent the compression properties, CE (compression energy) is the most important parameter.

**Figure 17:**
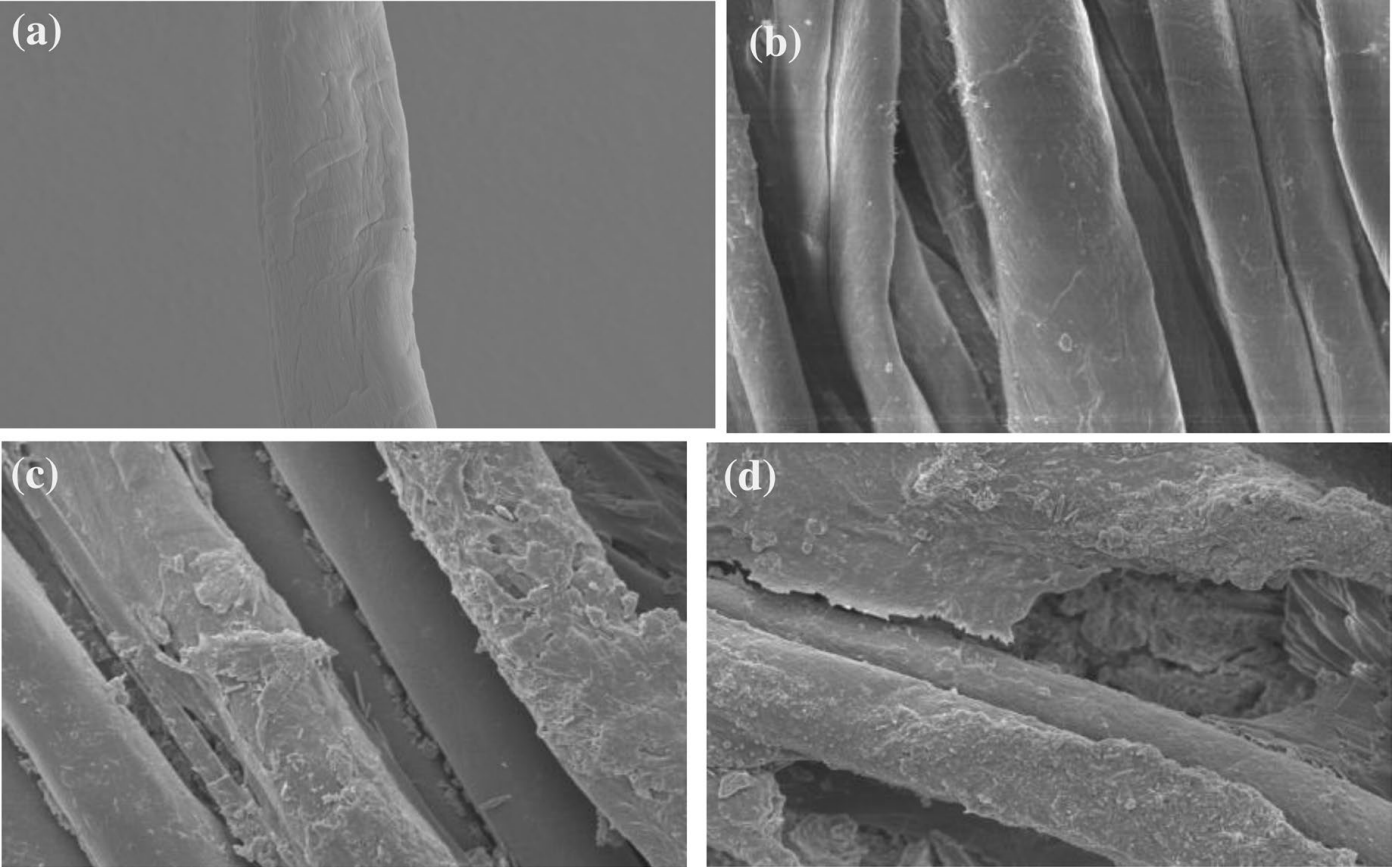
(**a**) Scanning electron microscopic image of 100% cotton control fabric. (**b**) Scanning electron microscopic image of 65/35 micro-denier polyester/cotton control fabric. (**c**) Scanning electron microscopic image of 65/35 micro-denier polyester/cotton sericin and glutaraldehyde finished fabric. (**d**) Scanning electron microscopic image of 65/35 micro-denier polyester/cotton (plasma-treated sericin and glutaraldehyde finished) fabric.

Figure 13 shows that with the exception of plasma- and sericin-glutaraldehyde-treated fabric (Sample 5), the linearity of the compression values are found to be the same for all the samples.When the compression force acts on the substrate's surface, protrude fibers may resist the load. When the load acts on the fiber, yarns come into close contact and are flattened and straightened, at which point inter-yarn and inter-fiber friction, as well as the yarn's bending stiffness, provide compression resistance until all fibers are in contact with one another^26^. From the SEM image in Fig. 17d the plasma- and sericin-glutaraldehyde-treated fabric showing more uniform surface coating on the fibers, so the distribution of the loadis resisting very high when compared to other samples, and similar trend we observed in the compressional energy values represented in Fig. 14.

Figure 15 shows the compressional resilience of the treated samples. With the exception of the glutaraldehyde treated sample (sample 2), there was a significant improvement in other cases. The reason for the low compressibility may be attributed to the stiffening effect. This can be attributed to the reduction in the mechanical restraint in the polyester fabric due to sericinand glutaraldehyde finishing. Morooka et al.^27^concluded that a combination of CE and RC can reflectthe softness of the fabric. Higher CE values accompanied by lower RC values generally lead to a softer fabric.

In Fig. 16, the percentage compression, which is a nonstandard parameter, shows a significant difference insamples 2, 3, and 5, glutaraldehydetreated sample, sericinand glutaraldehydetreated fabric and plasma- and sericinglutaraldehydetreated fabric respectively.

### Surface properties

Surface properties are related to the smoothness of fabrics and are represented by MIU, MMD and SMD. MIU is the ratio of the average frictional force to the normal load. MMD (mean deviation of MIU) gives the variability in the MIU value along with the fabric. The geometrical roughness (SMD) gives the average vertical displacement of the piano wire tip with reference to the mean surface layer of the fabric.

**Figure 18:**
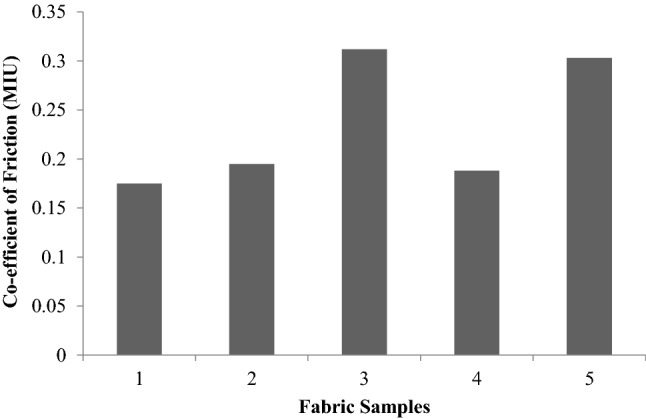
Co-efficient of friction (MIU) of samples.

**Figure 19:**
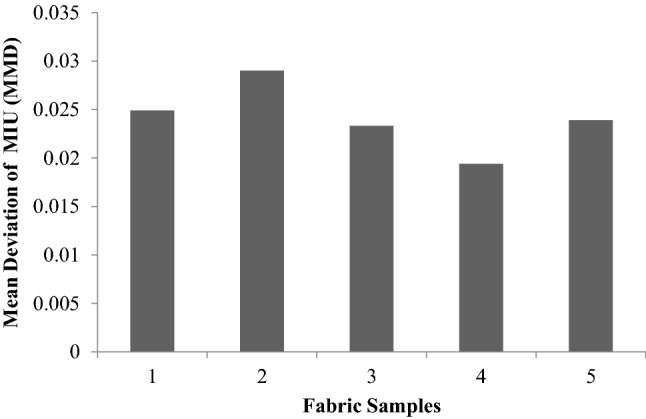
Mean deviation of MIU (MMD).

At a magnification of 5000, Fig. 17a offers a clear image of individual fibres in a 100% cotton fabric. Figure 18, shows that the MIU value is high in the case of sericin and glutaraldehyde samples and the case of plasma and sericin cum glutaraldehyde samples shown in Fig. 17c,d when compared to control represented in Fig. 17b. The increase in friction is due to the increase in the area of contact caused by the presence of sericin and glutaraldehyde. Figure 19 shows the mean deviation of MIU (MMD) and does not show any significant difference.

**Figure 20:**
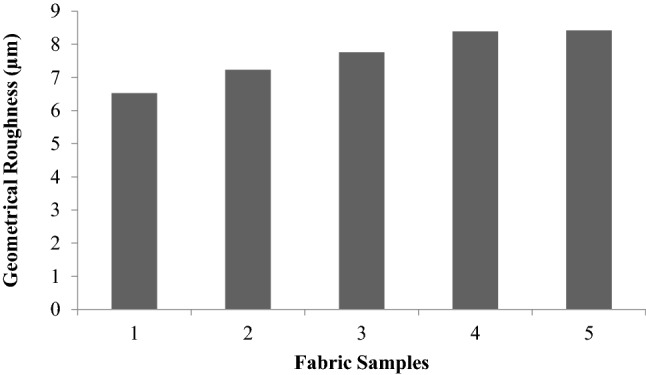
Geometrical roughness (SMD) of samples.

The variation in fabric surface roughness is depicted in Fig. 20. With the exception of glutaraldehydetreated fabric, all the other samples show an increase in surface roughness. The surface roughness increases with the glutaraldehydetreated sample, sericin and glutaraldehyde finish and plasma finished with sericin and glutaraldehyde samples compared to the control sample.

### Evaluation of fabric hand

Tables 2 and 3 shows the primary hand values (PHVs) and total hand values (THVs) for the textiles.The sensation of contact of fabric, which is relevant to the garment handle properties is the sensory comfort of garments. The subjective evaluation of textiles based on the sensation of touch has already been termed fabric handling. Fabric itchiness is determined by the diameter of fibers, fabric density at higher and lower pressures, and garment surface finish. The plasma-treated sample aids in the absorption of additional moisture, and the presence of moisture on the skin surface increases the intensity of fabric roughness sensations due to frictional changes.It is apparent that the plasma-treated sample displays a higher value, and the other values are comparable.

**Table 2:** Primary Hand Values.

Fabric samples/hand values	1	2	3	4	5
Koshi (Stiffness)	5.64	5.52	5.78	5.73	5.99
Numeri (Smoothness)	3.64	3.62	3.16	4.09	3.28
Fukurami (Fullness and softness)	4.27	4.11	4.12	4.68	4.50

*Sample 1—Control, Sample 2—Glutaraldehyde treated, Sample 3—Sericin and glutaraldehyde treated, Sample 4—Plasma treated, Sample 5—Plasma and sericin and glutaraldehyde treated.

**Table 3:** Total hand values.

Fabric samples	Total hand value
Control	2.80
Glutaraldehyde	2.70
Sericin and glutaraldehyde treated	2.75
Plasma treated	2.91
Plasma and sericin and glutaraldehyde treated	2.78

**Figure 21:**
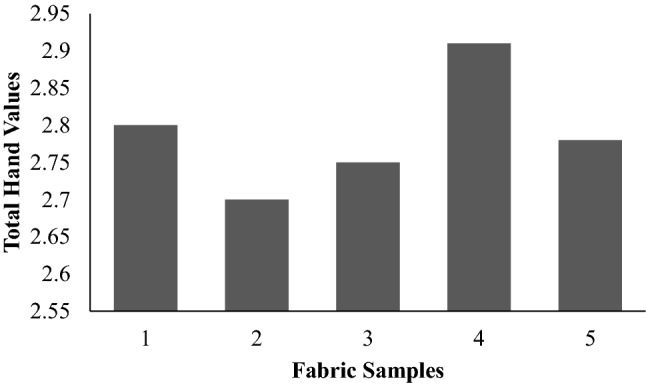
Total hand value.

In Fig. 21 all the treated samples are not very much different from the control which shows that there is no measurable difference in handle following the treatments given to them. In other words, there was no deterioration in the handling of micro denierpolyester fabric due to the treatments.

### ANOVA analysis of various factors

ANOVA was carried out for low-stress mechanical properties between treated and untreated fabrics. The results are given in Table 4, and it shows that there is no significant difference between various properties. However, between the treatments,there was a significant difference.

**Table 4:** Two-way ANOVA for low stress mechanical properties.

Source of variation	Sum of squares	DF	Mean sum of square	‘F’ value	‘p’ value
Between various properties	65,301.14	22	2968.234	1093.859	0.000*
Between treatment	3.248648	4	0.812	0.299	0.878^NS^
Residual	238.7918	88	2.714		
**Total**	65,543.19	114			

## Conclusion

The results show that the treatments led to significant differences in tensile resilience. The fabric on which sericin was applied following treatment with plasma first and thereafter with glutaraldehyde shows the lowest tensile resilience. There was no difference in the bending rigidity of the treated sample. Shear rigidity showed a significant increase in fabric treated with plasma cum sericin and glutaraldehyde. Shear hysteresis follows the same trend. Shear recovery was found to be poor in glutaraldehyde-treated samples, sericin-glutaraldehyde-treated samples and plasma-sericin-glutaraldehyde-treated samples. Compressional resilience was found to be very low in plasma cum sericin- and glutaraldehydetreated fabric and % compression also followed the same trend. The coefficient of friction was found to be higher in the plasma-sericin-glutaraldehyde-treated sample, and the mean deviation did not show any difference. Geometrical roughness shows a significant increase in sericin- and glutaraldehyde-treated, plasma-treated and plasma cum sericin- and glutaraldehyde-treated fabric samples in comparison to the control. There were no changes in fabric thickness, weight or porosity. As far as handle values of the samples are concerned, they are found to be the same.
